# Seasonal Patterns of Invasive Pneumococcal Disease

**DOI:** 10.3201/eid0905.020556

**Published:** 2003-05

**Authors:** Scott F. Dowell, Cynthia G. Whitney, Carolyn Wright, Charles E. Rose, Anne Schuchat

**Affiliations:** * International Emerging Infections Program, Bangkok, Thailand; † Centers for Disease Control and Prevention, Atlanta, Georgia, USA

**Keywords:** *Streptococcus pneumoniae*, seasons, pneumonia, weather, infection, communicable disease, temperature, photoperiod, child, research

## Abstract

Pneumococcal infections increase each winter, a phenomenon that has not been well explained. We conducted population-based active surveillance for all cases of invasive pneumococcal disease in seven states; plotted annualized weekly rates by geographic location, age, and latitude; and assessed correlations by time-series analysis. In all geographic areas, invasive pneumococcal disease exhibited a distinct winter seasonality, including an increase among children in the fall preceding that for adults and a sharp spike in incidence among adults each year between December 24 and January 7. Pneumococcal disease correlated inversely with temperature (*r* –0.82 with a 1-week lag; p<0.0001), but paradoxically the coldest states had the lowest rates, and no threshold temperature could be identified. The pattern of disease correlated directly with the sinusoidal variations in photoperiod (*r* +0.85 with a 5-week lag; p<0.0001). Seemingly unrelated seasonal phenomena were also somewhat correlated. The reproducible seasonal patterns in varied geographic locations are consistent with the hypothesis that nationwide seasonal changes such as photoperiod-dependent variation in host susceptibility may underlie pneumococcal seasonality, but caution is indicated in assigning causality as a result of such correlations.

As with many infectious diseases, the incidence of invasive pneumococcal infection rises and falls in an annual seasonal pattern that has been repeatedly documented but never well explained. The winter increase in cases has been attributed to cold weather, lower humidity, the crowding together of susceptible hosts, associated viral infections, and air pollution (*1*–*3*). Explanations for the cause of pneumococcal seasonality often appear contradictory. A temporary increase in the incidence of invasive disease was associated with two severe winters in the Netherlands (*4*), but in Alaska, the number of invasive cases is highest in summer months (*5*). The seasonal variation has been more apparent in adult invasive pneumococcal disease than in pediatric cases (*1*), although some seasonal variation in acquisition of nasopharyngeal colonization also has been documented in children (*6*). These disparate observations have been difficult to reconcile with a unifying explanation for pneumococcal seasonality.

A recent hypothesis proposes that the seasonal variation in the incidence of some infectious diseases is attributable to seasonal variation in host physiology, such as the density of a cellular receptor or the activity of the immune response (*7*). Many mammals exhibit distinct physiologic changes with the changing seasons, typically timed to the light-dark cycle and mediated by melatonin (humans exhibit some such changes) (*8*–*11*). Experiments with mice provide some evidence that susceptibility to fatal pneumococcal disease varies with the animal’s innate circadian rhythm (*12*–*14*). Determining the relevance, if any, of these observations to human pneumococcal infections would be advanced by a detailed description of the seasonal patterns of pneumococcal infections of various age groups and different geographic areas with distinct weather patterns. We evaluated data from a population-based surveillance system in seven geographic areas in the United States (Active Bacterial Core Surveillance) to describe the seasonal variation in invasive pneumococcal disease and explore these hypotheses.

## Methods

**Figure 1:**
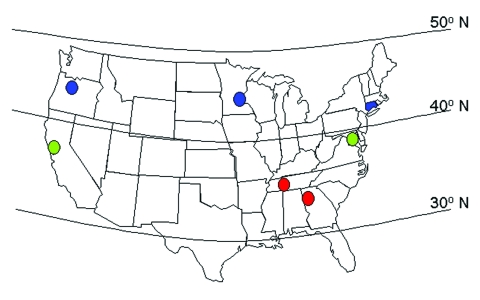
Map of the continental United States showing the approximate locations of the seven surveillance sites, grouped for some analyses as southern sites (illustrated in red), middle sites (green), and northern sites (blue).

Surveillance for invasive pneumococcal disease was performed from January 1, 1996, through December 31, 1998, in the counties of seven U.S. states, as consistently defined in the Active Bacterial Core Surveillance of the Emerging Infections Program Network. Geographic location was grouped by state and by latitude (Figure 1), including eight counties in Georgia and five counties in Tennessee (latitude 33°–35° north; defined here as southern sites), one county in California and six counties in Maryland (latitude 37°–39° north; middle sites), and the entire state of Connecticut, seven counties in Minnesota, and three counties in Oregon (latitude 41°–45° north; northern sites). Invasive disease caused by group A streptococci and group B streptococci was also assessed for comparison with pneumococcal disease.

Invasive disease was defined as disease in which an organism had been isolated from a normally sterile site (such as blood or cerebrospinal fluid) in a resident of the surveillance area. Each case was considered to have occurred on the date the culture was obtained. In each surveillance area, project personnel communicated regularly with contacts in all microbiology laboratories serving acute-care hospitals and completed standardized case-report forms. Audits were performed at least every 6 months to evaluate reporting sensitivity and identify unreported cases. All isolates were sent to the Centers for Disease Control and Prevention or the University of Texas Health Science Center at San Antonio on blood agar slants and confirmed as pneumococci on the basis of optochin susceptibility and bile solubility.

Daily surface weather data were obtained from weather stations in each surveillance site (Atlanta, Memphis, San Francisco, Baltimore, Hartford, Minneapolis, and Portland) from the National Climate Disease Center, Asheville, North Carolina (http://www.ncdc.noaa.gov). Sunrise and sunset times for each surveillance week were obtained for each of the above seven sites from the U.S. Naval Observatory, Washington, D.C. (http://aa.usno.navy.mil). Monthly figures for electric utility gas consumption were obtained from the Energy Information Administration, Washington, D.C. (http://www.eia.doe.gov), and figures for public construction expenditures were obtained from the U.S. Census Bureau, Washington, D.C. (http://www.census.gov).

Weekly numbers of cases were converted to annualized rates by dividing by the census population in the relevant counties for the appropriate surveillance year and multiplying by 100,000 population and 52 weeks (to get cases per 100,000 population per year). We then plotted annualized rates by state, latitude group (as categorized above), and age (adults >18 years and children).

Time series analysis was performed with SAS software (version 8.2, SAS Institute, Inc., Cary, NC) and Epi Info software version 6.02 (CDC, Atlanta, GA). Annualized weekly rate correlations with mean weekly temperature (in degrees F), total weekly precipitation (100ths of inches), and mean weekly minutes of darkness were calculated as Pearson correlation coefficients with time lags ranging from 0 to 8 weeks. Time lags were limited to an 8-week range because the highest correlations from exploratory analysis in the annualized weekly rate with the climatic data occurred within 2 months. Correlations with monthly gas consumption and construction expenditures were explored with time lags of 20 to 36 months because the data were available from 1999 to 2001, whereas the weekly pneumococcal disease rates were available from 1996 to 1998. Weather data were missing from Atlanta for 1996 and were not included; occasional missing readings were otherwise replaced by interpolation.

## Results

**Figure 2:**
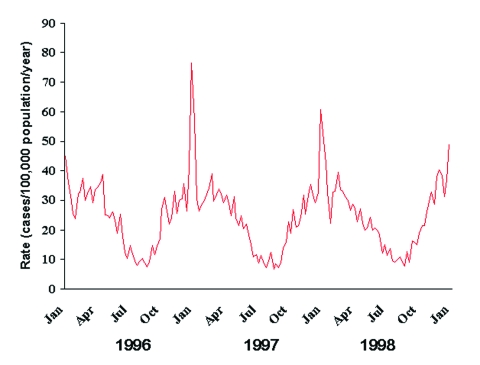
Weekly rates of invasive pneumococcal disease in the United States, January 1996–December 1998. Weekly numbers of cases from active surveillance areas in California, Connecticut, Georgia, Maryland, Minnesota, Oregon, and Tennessee were divided by the population under surveillance that year and multiplied by 52 to give annualized weekly rates.

Over the 3-year period, 11,614 cases of invasive pneumococcal disease were identified among the approximately 15,221,605 residents of the surveillance areas. The annualized weekly rate of invasive pneumococcal disease was distinctly seasonal, varying from approximately 10 cases per 100,000 population during the summer to approximately 35 cases per 100,000 during the winter (Figure 2). No seasonal variation was seen in the incidence of group B streptococcal infection, and a mild spring peak was seen in group A streptococcal infection. A prominent spike in the rate of invasive pneumococcal disease to 50–75 cases per 100,000 occurred during the last week of December and the first week of January in each of the surveillance years.

**Figure 3:**
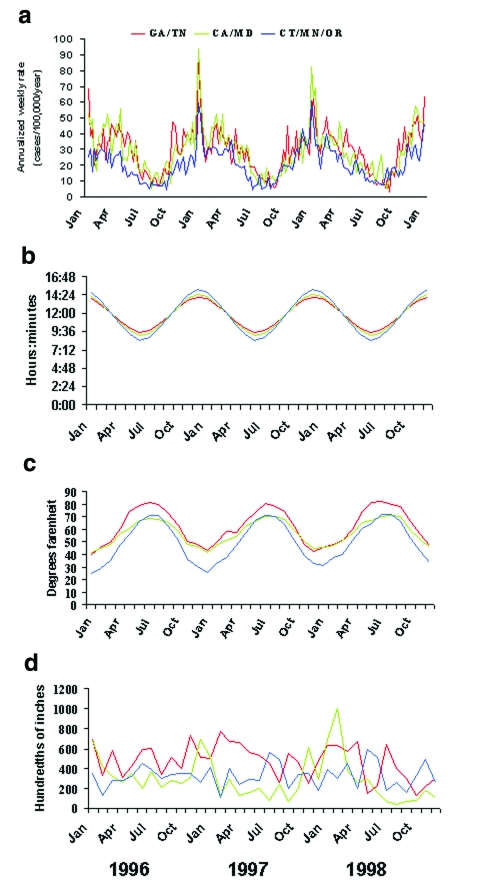
The relationship of invasive pneumococcal disease to photoperiod, temperature, and precipitation in three surveillance areas, grouped by latitude, 1996–1998. Southern surveillance areas were in Georgia and Tennessee (red lines; 33°–35° north latitude), middle areas were in California and Maryland (green lines; 37°–39° north latitude), and northern areas were in Connecticut, Minnesota, and Oregon (blue lines; 41°–45° north latitude). a: Rates of invasive pneumococcal disease; b: hours of darkness calculated for the 15th day of each month; c: mean monthly surface air temperature recorded at weather stations in each of the seven surveillance sites. d: total monthly precipitation recorded at weather stations in each of the seven surveillance sites. Pneumococcal disease correlated directly with photoperiod (*r* 0.85 with a 5-week lag; p<0.0001), indirectly with temperature (*r* –0.82 with a 1-week lag; p<0.0001), and poorly with precipitation (*r* 0 to <0.3).

The seasonal patterns were similar when the data from each state were plotted separately and when the states were grouped into the three latitude groups (Figure 3). The number of weekly cases increased approximately fourfold from summer to winter, and a prominent spike in the number of weekly cases occurred during the last week of December and the first week of January.

**Figure 4:**
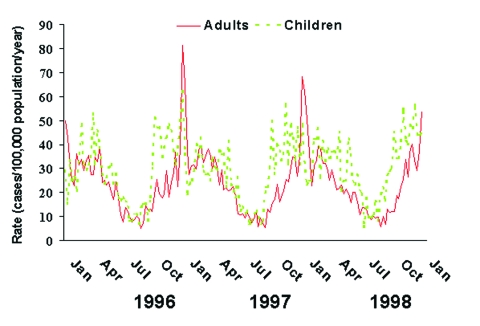
Weekly rates of invasive pneumococcal disease in children (dotted line; ages 0–17 years), and adults (solid line; age >18 years) in the United States, 1996–1998. Weekly numbers of cases from seven active surveillance areas were divided by the age-specific population and multiplied by 52 to give annualized weekly rates.

The pattern of seasonal variation was somewhat different in children compared to adults (Figure 4). The annual rise in incidence among children preceded that of adults, reaching the highest incidence during September and maintaining a high incidence throughout the fall. The spike in incidence during the last week of each year and first week of the subsequent year was seen in adults but not in children.

We further explored the characteristics of persons with invasive pneumococcal disease during the prominent annual spike (defined as December 20–January 10 of each year) and those experiencing invasive disease during the surrounding weeks (December 1–19, and January 11–31). Patients with invasive disease occurring during the spike and nonspike periods had similar demographic characteristics, except that adults were disproportionately represented during the spike (Table). After children were excluded, the median age of adults with invasive disease during the spike was 60 years, compared with 56 years for adults in the surrounding weeks (p=0.05). No similar spike in incidence occurred for either group A or group B streptococcal disease.

**Table:** Characteristics of persons with invasive pneumococcal disease during an annual winter spike in incidence compared with those experiencing invasive pneumococcal disease during the surrounding weeks, 1996–1998.^a^

Characteristic	Percentage of patients with characteristic		p value^b^
	Nonspike (N=1,647)	Spike (N=1,351)	
Sex (% male)	54.9	51.4	0.07
Race (% white)	56.6	60.8	0.07
Age (% adult)	74.3	81.7	0.0000013
Survival	87.1	87.5	0.92
State			0.49
California	6.0	4.9	–
Connecticut	17.7	18.9	–
Georgia	25.6	25.3	–
Maryland	20.2	19.1	–
Minnesota	10.4	11.3	–
Oregon	6.8	5.8	–
Tennessee	13.3	14.7	–
Syndrome			0.24
Bacteremia	35.6	33.0	–
Pneumonia	56.4	59.6	–
Meningitis	4.9	4.6	–

^a^The spike period was defined as December 20–January 10 of each year, and the nonspike periods were December 1–19 and January 11–31. ^b^p value calculated by chi-square test of the 2 x N table, with Yates correction.

The seasonal peaks in invasive pneumococcal disease in each of the three latitude groups correlated with increases in the number of hours of darkness and with cold temperatures in the winter, but correlation was poor with seasonal variations in total precipitation (Figure 3). Correlations were highly significant (Pearson’s correlation coefficients all < –0.5; p<0.0001) for pneumococcal disease and temperature, with time lags ranging from 0 to 8 weeks (highest *r* –0.82 with a 1-week lag). Correlations were also highly significant (*r* all >0.7; p<0.0001) for minutes of darkness and pneumococcal disease (highest *r* +0.85 with a 5-week lag). Temperature and photoperiod were themselves highly correlated with each other (*r* –0.95; p<0.0001). Precipitation did not correlate well with pneumococcal disease (*r* all <0.3, with best correlation of 0.29 using a 0-week lag).

The temperature curves for the individual states showed distinct patterns (data not shown), as did the temperature curves when sites were grouped by latitude (Figure 3c), but these variations were not associated with site-specific variation in pneumococcal disease. For example, the northern sites, with temperatures averaging 10°–20° lower than the southern sites throughout the year, did not have higher rates of invasive pneumococcal disease (Figure 3a). No evidence of a longer pneumococcal season in northern sites than in southern sites was evident, a finding that might have been expected if a threshold temperature existed, below which pneumococcal disease rates increase.

**Figure 5:**
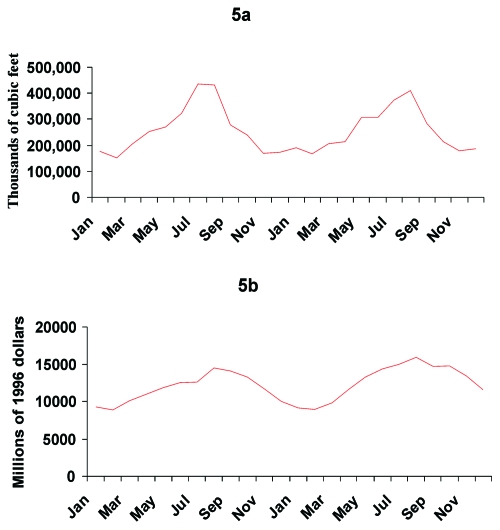
Seasonal variations in U.S. electric utility gas consumption (a) and public construction expenditures (b). Both parameters correlated indirectly with the invasive pneumococcal disease rates (gas consumption: *r* –0.92 with a 22-month lag; and construction: *r* –0.84 with a 35-month lag). Data were obtained from the Energy Information Administration and the U.S. Census Bureau, respectively.

Seasonal variations in photoperiod, or the length of the light-dark period, also corresponded with the seasonal variation in pneumococcal disease. Photoperiod was a seasonally varying phenomenon with less site-to-site variation than temperature (Figure 3b), similar to the consistent pattern of pneumococcal disease rates across the sites. The variation in the magnitude of the photoperiod curves was slightly more pronounced for northern sites than for southern sites (Figure 3b), a pattern which was not seen in invasive pneumococcal disease (Figure 3a). Associations between the seasonal increases in invasive pneumococcal disease and seemingly unrelated phenomena were not difficult to demonstrate, such as the correlations with seasonal variation in public construction expenditures (*r* –0.84 with a 35-month lag) and electric utility gas consumption (*r* –0.92 with a 22-month lag) (Figure 5).

## Discussion

A distinctive and regular seasonal variation in the incidence of invasive pneumococcal disease was confirmed by this analysis, as has been reported by others (*1*,*3*,*15*–*17*). We found that the patterns of seasonal variation were remarkably consistent across our seven geographically distinct surveillance areas, and we used the site-specific differences in weather across these areas to evaluate proposed explanations for the cause of pneumococcal seasonality. The unique seasonal patterns in children’s infections as compared to those of adults, and the peculiar spike in incidence in adults during the same 1- to 2-week period each year remain incompletely explained, although these observations should provide ample opportunities for testing hypotheses in future studies.

The shapes of the invasive pneumococcal disease curves were remarkably consistent across all seven surveillance sites, including the timing of the summertime nadir, the fall upswing in incidence, and the midwinter peak. These patterns were consistent despite marked differences in rainfall patterns and, to a lesser extent, differences in air temperature across these geographically widespread sites. If colder temperature is what drives the winter increase in pneumococcal disease, we might have expected higher rates throughout the year from northern sites, but that is not what we found. Alternatively, pneumococcal disease might increase when temperatures drop below some biologic threshold. Such biologic thresholds are present in certain temperature-sensitive viruses, for example, that are useful as vaccines because they are not viable in the warmer temperatures of the human lung (*18*). Such a temperature threshold for pneumococcal disease might be expected to result in a longer “pneumococcal season” in northern sites, a pattern we also did not find.

Temperature and photoperiod variations need to be investigated further with datasets obtained by using similar methods from regions with wider latitude and temperature variations than we used here. Preliminary review of a smaller dataset of invasive pneumococcal disease from a different surveillance system in Alaska did not identify elevated rates of pneumococcal disease associated with the colder weather and longer winter and also did not display the consistent seasonal variations seen in our continental U.S. sites (J. Butler, pers. comm.).

Some of the apparent variation in incidence of invasive pneumococcal disease seen in different studies may be attributable to variations in blood-culturing patterns (*19*,*20*). Geographic variation in blood culture practices may be more likely to influence the size of seasonal peaks and less likely to affect their timing, as we observed. Strengths of this analysis include the large population under surveillance and the consistent methods used to identify cases across all seven geographically distinct surveillance areas; these factors should tend to minimize any effects of local differences in blood-culturing practices or institutional variation in case ascertainment.

The similar seasonal patterns observed in our seven surveillance sites, despite their wide variations in air temperature, precipitation, and other weather patterns, argue that the signal guiding pneumococcal seasonal variations is one that is more consistently present in all sites. Photoperiod has been shown to be the most pervasive signal for seasonal changes in biologic systems (*10*,*21*–*23*), and we believe our findings are consistent with the proposal that photoperiod also contributes to the seasonality of invasive pneumococcal disease in humans. The sinusoidal variation in photoperiod across the sites correlated closely with the pattern of invasive pneumococcal disease. We did not find a greater magnitude of pneumococcal variation from northern sites, and definitively identifying photoperiod, rather than temperature, as the driving phenomenon was not possible from this dataset. Because temperature and photoperiod are themselves highly correlated, evaluating the relative contributions of each to invasive pneumococcal disease will likely require further exploration with more years of data from more geographically diverse sites.

We emphasize that correlating seasonal variations in invasive pneumococcal disease with temperature and photoperiod provides insufficient evidence to establish a causal link. Using brief searches of the Internet, we readily identified seasonal variations in housing construction, gas consumption, and other seemingly unrelated phenomena that were also significantly correlated with the seasonal variation in pneumococcal disease. Because so many phenomena vary seasonally, evaluating one or two hypothesized causes for seasonal variation in disease and concluding that the close correlation supports causality is a pitfall all investigators in this field must take care to avoid.

Accumulating evidence is supporting the biologic plausibility that preceding respiratory virus infections, influenza in particular, increase susceptibility to invasive pneumococcal disease (*24*,*25*). We did not have comparable data on virologically confirmed influenza infections from each of the seven surveillance sites to include in this analysis, but such information should be sought in future evaluations.

Some experimental evidence exists to support the concept that cyclical variation in susceptibility to pneumococcal infections is present in the mammalian host, and that the light-dark cycle provides the signal that entrains this cyclical pattern. Mice kept in windowless rooms with a controlled light-dark cycle are more susceptible to pneumococcal infection during the dark phase (*12*). When such mice were inoculated intraperitoneally with virulent pneumococci during the dark phase (0400), there was a slower increase in bacteremia (*13*), and the mice survived significantly longer, compared to those challenged during the light phase (*12*). In blinded mice, this pattern of decreased susceptibility was maintained on an approximately 24-hour (circadian) pattern but cycled out of phase with the room lighting. These findings indicate the presence of an endogenous rhythm of susceptibility to pneumococcal infection that is normally entrained to a strict 24-hour pattern by visual detection of the daily light-dark cycle (*14*). Whether this circadian variation in susceptibility is accompanied by seasonal variation has not been evaluated, although the two periodic phenomena are closely related in most biologic systems (*23*). Humans retain the physiologic capacity to respond to changes in photoperiod (*11*,*26*–*28*), but some evidence exists that artificial lighting blunts these responses (*29*). Moreover, the degree to which seasonal changes in light cycles influences human physiology, if at all, is not clear.

The seasonal pattern in children was distinct from that in adults, consistent with differences in clinical disease in children and with the observations of others (*1*,*5*,*30*,*31*). Children are the reservoirs for pneumococci in the community, with nasopharyngeal colonization varying slightly (*6*), if at all (*32*), throughout the year. We observed a seasonal peak in children that was broader and flatter than that in adults, with an early autumn rise to the peak and an absence of the midwinter spike. Others have described this as a biphasic pattern, with a flattening or drop in incidence in midwinter (*30*,*31*,*33*,*34*); we did not observe this pattern. We speculate that the early autumn rise may be associated with the return to school and exchange of new serotypes, which may then be transmitted to adult contacts. The substantial variation in invasive disease despite fairly steady carriage supports a role for increasing host susceptibility or other predisposing factors, rather than appearance and disappearance of the pathogen, as an underlying explanation.

The prominent midwinter spike in incidence we observed does not appear to be an artifact of the surveillance system. The spike, present in all seven states, was not seen for group A or group B streptococcal disease, which were reported through the same system. Persons with invasive disease during the spike were significantly older but otherwise demographically similar to those with invasive disease during the surrounding weeks. The consistent timing of the spike during the weeks of December 24–January 7 is provocative. This is a time when many U.S. families gather for Christmas and New Year’s holidays, perhaps providing an opportunity for exposure of older relatives to new serotypes from young children at a time when their annual susceptibility to pneumococcal disease is at its peak. Previous studies have documented an association between exposure to young children and invasive pneumococcal disease (*35*,*36*). Others have not reported such a spike, but most such analyses have grouped the data by month, an approach that would have led us to miss the spike in our data.

We encourage others with similar databases to examine their data by weekly intervals, to distinguish patterns in adults from those in children, and to report information on latitude, temperature patterns, humidity, and other variables to allow for a consistent exploration of the influences on pneumococcal seasonality. Variation in susceptibility in the human host is one hypothesis that should be routinely considered.
